# Enhancing and promoting data management and systematic monitoring for an improved HIV/AIDS programs in Addis Ababa, Ethiopia

**DOI:** 10.1186/s12913-021-07442-9

**Published:** 2022-01-09

**Authors:** Dereje Habte, Samuel Zemenfeskudus, Mulugeta Endale, Mohammed Zeidan, Daniel Getachew, Dejene Woldemichael, Aklilu S. Wesene, Esayas Teklebirhan, Fitsum Eyayu, Raey Zewdie, Daniel Yirga, Worknesh Amdino, Zenebe Melaku, Sisay A. Abayneh

**Affiliations:** 1 The United States Centers for Disease Control and Prevention, Division of Global HIV & TB, Addis Ababa, Ethiopia; 2 Addis Ababa City Administration Health Bureau, Addis Ababa, Ethiopia; 3 ICAP at Columbia University, Addis Ababa, Ethiopia

**Keywords:** Human immunodeficiency virus (HIV), HIV testing, Anti-retroviral treatment, HIV program, Monitoring and evaluation, Data use, Information use, Addis Ababa

## Abstract

**Background:**

Ethiopia Population-based HIV Impact Assessment findings showed that in Addis Ababa, only 65.2% of people living with HIV (PLHIV) know their status. We present the enhanced HIV/AIDS data management and systematic monitoring experience in Addis Ababa City Administration Health Bureau (AACAHB).

**Methods:**

AACAHB established a command-post with leadership and technical team members from the health bureau, 10 sub-city health offices, and non-governmental stakeholders. The command-post improved governance, standardized HIV program implementation, and established accountability mechanism. A web-based database was established at each health facility, sub-city, and AACAHB level. Performance was scored (green, ≥75%; yellow, 50–74%; red, < 50%). The command-post reviewed performance on weekly basis. A mentorship team provided a weekly site-level support at underperforming public and private health facilities. At facility level, quality of data on recording tools such as registers, and individual medical records were maintained through continued review, feedback mechanisms and regular consistency check of data. Percentage and 95% confidence interval were computed to compare the improvement in program performance over time.

**Results:**

After 6 months of intervention period, the monthly New HIV case finding in 47 health facilities increased from 422 to 734 (1.7 times) and treatment initiation increased from 302 to 616 (2 times). After 6 months, the aggregate scoring for HIV testing at city level improved from yellow to green, HIV case finding improved from red to green, and treatment initiation improved from red to yellow. An increasing trend was noted in HIV positive case finding with statistically significant improvement from 43.4% [95% Confidence Interval: 40.23–46.59%] in May 2019 to 74.9% [95% Confidence Interval: 72.03–77.6%] in September 2019. Similarly, significant improvement was recorded for new HIV treatment from 30.9% [95% Confidence Interval: 28.01–33.94%] in May 2019 to 62.5% [95% Confidence Interval: 59.38–65.6%] in September 2019.

**Conclusions:**

Regular data driven HIV program review was institutionalized at city, sub-city and health facility levels which further improved HIV program monitoring and performance. The performance of HIV case finding and treatment initiation improved significantly via using intensified monitoring, data driven performance review, targeted site-level support based on the gap, and standardized approaches.

## Background

Ethiopia has made substantial progress in addressing the HIV epidemic and there is a plan to further strengthen HIV case finding, care, and treatment services [1, 2]. HIV prevalence in the adult population aged 15–49 years has declined steadily over the last decade among both women and men. The estimated national HIV prevalence in 2016 was 0.9%, with higher prevalence among women (1.2%) than men (0.6%). Ethiopia has a mixed epidemic with wide regional variations and concentrations in urban areas (urban HIV prevalence, 2.9%; rural, 0.4%) [3]. In 2019, Ethiopia had an estimated 606,933 people living with HIV (PLHIV), of whom 474,532 are receiving treatment, making Ethiopia a high HIV burden country [4].

In 2016, Addis Ababa city, the capital and largest city in Ethiopia, had an estimated 3.4% HIV prevalence, which is significantly higher than the national urban prevalence and the prevalence in many other regions in Ethiopia [3]. Spatial distribution of HIV cases in the three Ethiopian Demographic and Health Surveys (2005, 2011, and 2016) showed that Addis Ababa city and the surrounding areas continue to have the highest prevalence of HIV in Ethiopia [5]. In 2018, Ethiopia Population-based HIV Impact Assessment (EPHIA) findings showed that in Addis Ababa, HIV prevalence is 3.1% among adults aged 15–49 years and that only 65.2% of PLHIV are aware of their status, resulting in an estimated detection gap of 26,764 PLHIV to reach the first of the 90–90-90 targets established by the Joint United Nations Program on HIV/AIDS (UNAIDS): 90% of PLHIV know their status; of these, 90% are receiving antiretroviral therapy (ART); and of these, 90% have viral load (VL) suppression [6]. In Addis Ababa, EPHIA showed that only 63.3% of those who know their HIV-positive status were receiving ART, and the VL suppression rate was low (58.2%) [7].

To help meet the UNAIDS 90–90-90 targets, in April 2019, Addis Ababa City Administration Health Bureau (AACAHB) launched an HIV case finding and treatment linkage campaign to accelerate ART enrollment of PLHIV and increase ART coverage across all age groups. Our accelerated approach introduced new methods of data management and systematic monitoring, promoted active engagement of HIV program team in weekly performance monitoring, and enhanced evidence-based decision making at the health facility, sub-city, and regional levels. We present the enhanced data management and systematic monitoring which significantly improved HIV/AIDS program performance in Addis Ababa.

## Methods

### Setting

AACAHB administers the overall health system in Addis Ababa. There are 10 sub-cities under AACAHB with sub-city health offices to manage public and private health facilities in their jurisdiction. There are 11 government hospitals, 32 private hospitals, 99 public health centers, and 1143 private clinics providing comprehensive health service. A total of 93 public and 53 private health facilities providing HIV testing and treatment services were included in the systematic monitoring initiative. Two government hospitals have their own VL testing machine while the remaining public and private health facilities included in the systematic monitoring were referring blood sample to a VL testing laboratory located in Addis Ababa. The five VL testing laboratories in Addis Ababa serve designated health facilities, and samples are transported from the health facilities to the VL laboratory via the Post Office. HIV experts at AACAHB, sub-city health offices, and high HIV volume health facilities (over 300 patients on ART) coordinate HIV prevention, case finding, and treatment activities. Health workers in public and private health facilities provided comprehensive health services, including HIV/AIDS services.

Before the intervention, HIV/AIDS aggregate report used to reach AACAHB via the national health management information system (HMIS) on monthly and quarterly basis from the sub-city health offices. HMIS report was reviewed mainly by the monitoring and evaluation team at AACAHB, cleaned and further submitted to MOH. There had been no organized weekly or monthly performance review at AACAHB for any health program including HIV/AIDS. Health facility level HMIS performance review was not a standard practice before the intervention. The leadership at AACAHB managed all health program implementation at AACAHB including HMIS reporting but they were not actively engaged in data driven health program reviews regularly.

### Health system leadership, coordination, and standardization

AACAHB leadership spearheaded the accelerated approach by designing the coordination system and managing human, financial, and material resources at all levels. The accelerated approach refers to the whole set of interventions devised differently in intensifying the efforts for HIV case finding, treatment linkage, viral load testing and suppression. The accelerated interventions included active engagement of the leadership at all levels; coordinated effort among clinical, HIV program, laboratory, logistics, and monitoring and evaluation team; efficient use of existing human and financial resources; weekly review of HIV program performance; enhanced use of data driven decision making; provision of tailored support to poorly performing health facilities; standardization of HIV related service provision; and establishment of systematic monitoring mechanism. AACAHB organized a dedicated weekly leadership meeting in addition to the weekly technical review at the city level. The weekly leadership meeting identified issues for further action in relation to HIV case finding, treatment linkage, VL testing and administrative issues. AACAHB leadership provided guidance to the sub-cities and health facilities on the importance of the accelerated approach, how to address technical issues, and the monitoring mechanism.

Sub-city health office and health facility leadership cascaded the accelerated approach to their team and led the activity in their jurisdiction. Weekly and bi-weekly performance reviews were organized at health facility and sub-city levels, respectively. Multi-disciplinary teams including leadership, data managers, and experts in HIV, laboratory, and pharmacy attended meetings at the health facilities and sub-city levels to review performance and develop performance improvement plans. The leadership and technical team emphasized standard-based approaches in HIV program implementation, monitoring, and evaluation. A standardized approach was ensured via trainings, standard operating procedures, dedicated staff, regular site level support, and joint supportive supervision.

### Enhanced Monitoring and Evaluation (M&E) framework and central data center

**Fig. 1:**
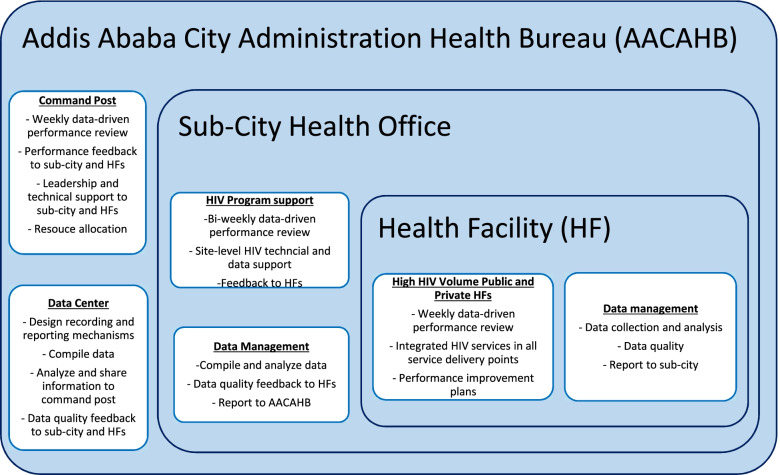
Monitoring and Evaluation Accelerate Progress Toward HIV Case Finding in Addis Ababa, Ethiopia (April–September 2019)

Routine HMIS data were compiled at the regional level on a quarterly basis. Health facilities aggregated their data monthly and reported to the sub-city health office quarterly [8]. AACAHB led a team including representatives from the regional health bureau, sub-city health offices, health facilities, and implementing partners to develop an enhanced M&E framework to address the data needed for the accelerated approach (See Fig. 1). The framework identified the levels of health facilities to be engaged; defined the indicators and hierarchy of reporting; established the frequency of indicator reporting as weekly or monthly; and determined the mechanism of data review and use at the health facility, sub-city, and city levels. AACAHB, sub-city health offices, and health facilities also identified dedicated HIV and M&E experts to support HIV program implementation, monitoring, and evaluation.

The AACAHB regional command post included representatives from senior management, the HIV program, M&E, laboratory, drug supply, sub-city, and implementing partners. The command post conducted weekly technical reviews, which were chaired by AACAHB leaders. A data center was established at AACAHB under the M&E department to lead data management, information sharing, monitoring, and evaluation. The data center worked closely with sub-cities and health facilities to oversee data collection, data quality, reporting, and data use at all levels. The data center compiled, analyzed, and shared data with the regional command post. The command post reviewed weekly performance for each sub-city and hospital in terms of the HIV testing, HIV positive, new HIV treatment and viral load testing targets to identify poor performance at all levels. Issues that needed the technical team’s attention were identified for immediate action, and the status of the action points were reviewed in subsequent meetings. Furthermore, AACAHB led two regional review meetings during the intervention period, a full day meeting, to disseminate improvement strategies, build skills regionally, improve local knowledge, and share successful experiences.

The performance monitoring team at each health facility included ART clinicians, data managers, M&E experts, pharmacists, laboratorians, and the medical director. The team reviewed data on weekly basis and evaluated progress toward the HIV testing, HIV positive, new HIV treatment, and VL testing targets allocated to each health facility. Key responsibilities included reviewing performance monitoring data, checking data quality, prioritizing areas for HIV service improvement, developing performance improvement plans, providing support to the health facility teams regularly, and coordinating team members. The performance monitoring team met weekly, recorded the outcome and action points of each meeting, and monitored the status of resource availability, HIV testing, treatment linkage, and VL testing. Ongoing work at health facilities was overseen via monthly visits by the regional or sub-city technical team.

At facility level, quality of data on recording tools such as registers, and individual medical records were maintained through continued review and feedback mechanisms and regular consistency check of data between medical records and registers. Reporting quality as measured by completeness (both content and representative), timeliness, reliability and validity were maintained through the continuous application of techniques such as visual scanning, consistency check via Lot quality assurance sampling (LQAS) techniques and community verification. The sub-city and city administration health bureau conducted regular report review and feedback, desk review of data quality status, routine data quality assessment, and community verification.

### HIV program monitoring platform

**Fig. 2:**
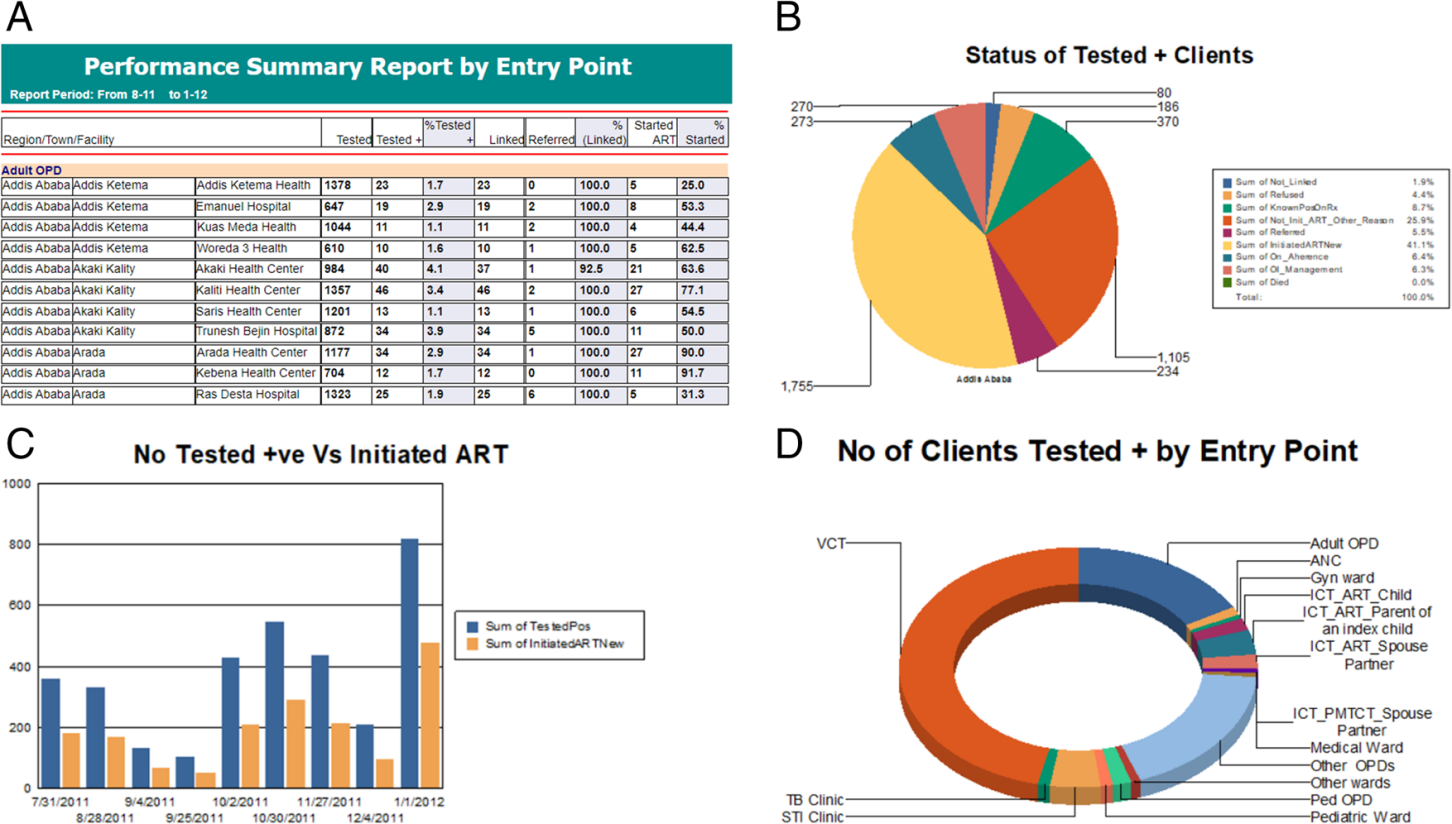
PTQIT Used to Accelerate HIV Case Finding in Addis Ababa, Ethiopia (April–September 2019)

The priority town quality improvement tool (PTQIT) was used in HIV program monitoring in high HIV volume facilities in selected towns in Ethiopia. Per the M&E framework, the web-based PTQIT software was upgraded for weekly and monthly data transmission from health facilities to AACAHB using state-of-the art object-oriented development tools. PTQIT had five main modules: Entry, Report, Data Exchange, Setting, and Downloads (See Fig. 2). Health facility staff were trained to use PTQIT software, the data capturing tool was provided to health facilities with high HIV volume (*n* = 47), and dedicated health facility M&E experts were assigned to lead HIV data management.

Health facility data were entered into the web-based system to facilitate review and data use at the health facility, sub-city, and AACAHB levels. PTQIT software captured facility-level summary data in relation to HIV testing, HIV test results, modalities of HIV testing, linkage to ART, VL testing, and VL test results. Moreover, the database also captured additional indicators for the supply chain and accomplishments of scheduled review meetings. HIV testing and treatment initiation data were reported on a weekly basis, and VL testing and the additional indicators are collected on a monthly basis.

Low HIV volume government health facilities (*n* = 46) and all private health facilities (*n* = 53) submitted paper-based reports to the sub-city health office for data entry into PTQIT software, and the data were transmitted to AACAHB. The sub-city and AACAHB M&E team supported data management and provided feedback to health facilities via the hierarchy in the health system.

### Data review and information use

**Fig. 3:**
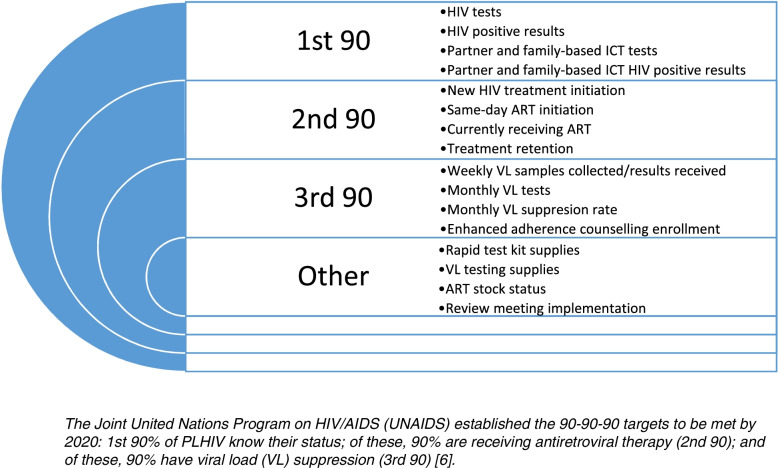
Indicators Used to Accelerate Progress Toward the Joint United Nations Program on HIV/AIDS 90–90-90 Targets in Addis Ababa, Ethiopia (April–September 2019)

The primary focus in the accelerated campaign was to clean data and promote data-driven decision making at the health facility and sub-city levels. The data center at AACAHB worked closely with the HIV program team to select indicators, develop the analysis plan, and facilitate information use for HIV program improvement. To facilitate the review of HIV service performance at all levels, AACAHB distributed performance targets to the respective health facilities and sub-cities pertaining to HIV testing, ART linkage, VL coverage, and VL suppression (See Fig. 3). Data were reviewed weekly at the health facility and regional level, and bi-weekly at the sub-city level. For HIV testing, positive test results, and VL coverage, the score range was categorized by color: green, ≥75%; yellow, 50–74%; and red, < 50%. The scoring range was determined by the monitoring and evaluation team based on experience where performance below 50% of the target was considered poor.

The regional and sub-city HIV experts used these scores to identify health facilities with poor performance and provided weekly tailored site-level support per the performance improvement plan. AACAHB senior management also organized a comprehensive monthly and quarterly performance review meeting at the regional level. Meeting participants included AACAHB experts, the sub-city team, the team from selected high HIV volume health facilities, and HIV program implementing partners. Regional and sub-city HIV experts conducted regular support visits at health facilities using standard paper-based mentorship checklist. Facilities with performance gaps developed performance improvement plans with support from regional and sub-city experts and were visited more frequently to monitor improvement following the interventions.

### Data analysis

Data collection started in May 2019 (baseline). Data presented at the regional level were disaggregated by sub-city and health facility to show the performance at lower levels and provide tailored support based on the gaps identified. The web-based database has inbuilt custom analysis tools and a dashboard for the clinical and program team to review performance and make decisions at the health facility and higher levels (See Fig. 2). Role-based access to PTQIT was granted to relevant staff at the health facility, sub-city, and AACAHB levels for data management and information use purposes. Dynamic reports incorporated in the system enabled evidence-based planning and interventions, and monitoring from the health facility level up to the regional level. Tableau software was used at AACAHB to present results and facilitate data review at the regional level [9].

In this paper, data were captured in the web-based system and exported into Microsoft Excel for frequency, percentages, and trend analysis. Percentage and 95% confidence interval were computed to compare the performance at baseline with the result after the intervention. Statistical significance is confirmed when there is no overlap in the 95% confidence interval between the two comparison results.

## Results

We present the data from 47 high HIV volume public health facilities in 10 sub-cities. HIV testing indicator score at baseline was green in three sub-cities while nine sub-cities scored green after 5 months. There was no sub-city with green score for HIV case finding indicator at baseline and, six sub-cities scored green after the intervention. Nine sub-cities had a red score and one had a yellow score for HIV treatment at baseline; after the intervention, 2 sub-cities scored green, and 8 achieved a yellow score. After the intervention, the city level scoring for HIV testing improved from yellow to green (1.9 times increase from baseline, 61.4 to 114.6%), HIV case finding improved from red to green (1.7 times increase from baseline, 43.4 to 74.9%), and treatment linkage improved from red to yellow (2 times increase from baseline, 30.9 to 62.5%) (See Table 1).

**Table 1:** Improved HIV case finding and treatment linkage scores by sub-city over time, Addis Ababa, Ethiopia (May–September 2019)

Sub-City	HIV Testing Performance (%)						HIV Positive Performance (%)						New HIV Cases Treatment Performance (%)					
	Monthly target	May 2019	Jun 2019	Jul 2019	Aug 2019	Sept 2019	Monthly target	May 2019	Jun 2019	Jul 2019	Aug 2019	Sept 2019	Monthly target	May 2019	Jun 2019	Jul 2019	Aug 2019	Sept 2019
**Addis Ketema**	1106	117.9	148.0	156.1	189.2	193.0	32	68.8	56.3	59.4	106.3	106.3	32	65.6	21.9	40.6	118.8	68.8
**Akaki Kality**	4654	31.8	41.6	54.4	74.6	66.1	136	33.1	55.1	40.4	48.5	69.9	136	28.7	36.8	36.0	33.1	61.8
**Arada**	2985	65.7	80.5	99.9	111.3	134.4	89	51.7	49.4	65.2	77.5	79.8	89	39.3	56.2	56.2	77.5	58.4
**Bole**	2495	51.7	85.9	102.4	121.1	110.4	75	41.3	50.7	50.7	58.7	62.7	75	25.3	37.3	44.0	46.7	52.0
**Gulele**	4283	64.9	75.6	92.4	111.2	124.6	119	42.6	52.0	54.5	61.5	74.8	119	26.4	41.2	35.7	36.4	53.8
**Kirkos**	4494	55.9	57.9	67.5	96.3	106.8	137	35.0	40.9	39.4	51.8	55.5	137	27.0	43.1	51.8	55.5	55.5
**Kolfe Keraniyo**	3890	52.6	62.6	70.2	87.3	95.0	119	34.6	39.1	44.4	48.7	61.3	119	18.8	24.1	40.6	38.7	50.4
**Lideta**	925	99.9	165.2	100.1	151.7	158.7	28	57.1	82.1	50.0	51.9	78.8	28	46.4	75.0	51.9	48.1	82.7
**NefasSilk- Lafto**	3795	81.9	86.4	79.9	129.5	129.4	106	44.3	38.7	50.9	61.3	80.2	106	31.5	29.7	43.2	54.1	65.8
**Yeka**	3017	68.4	64.6	80.4	85.5	122.4	88	65.9	44.3	55.7	57.8	115.4	88	44.3	44.3	52.3	49.4	98.9
**Total**	31,644	61.4	73.0	82.2	106.5	114.6	929	43.4	47.6	49.4	58.6	74.9	929	30.9	38.9	44.4	49.8	62.5

Performance was scored (green, ≥75%; yellow, 50–74%; red, < 50%)

**Fig. 4:**
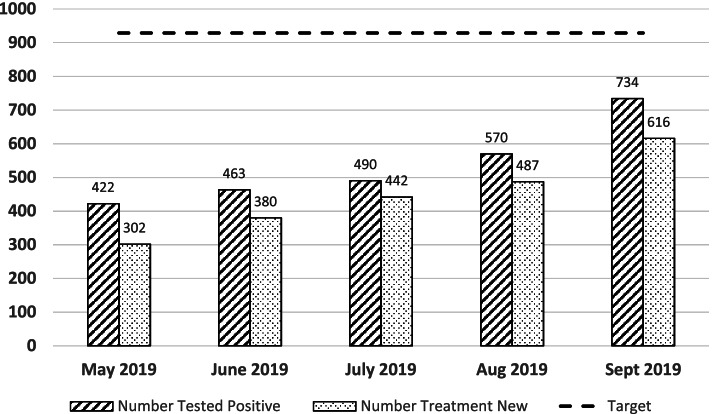
Monthly Performance of HIV Case Finding and ART Initiation at City Level, Addis Ababa, Ethiopia (May–September 2019)

**Fig. 5:**
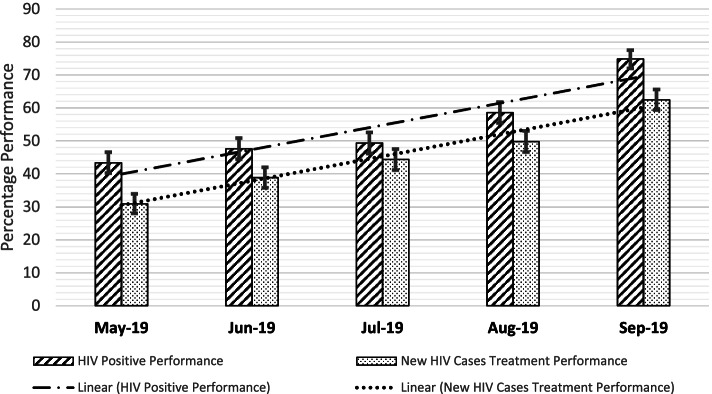
Increasing Trend in Percentage Performance of HIV Case Finding and ART Initiation at City Level, Addis Ababa, Ethiopia (May–September 2019)

In May 2019, 422 people with a new HIV diagnosis were identified and it increased to 734 in September 2019 (1.7 times). New ART initiation increased by two folds from 302 in May 2019 to 616 in September 2019 (See Fig. 4). An increasing trend was noted in HIV positive case finding with statistically significant improvement from 43.4% [95% Confidence Interval: 40.23–46.59%] in May 2019 to 74.9% [95% Confidence Interval: 72.03–77.6%] in September 2019. Similarly, significant improvement was recorded for new HIV treatment from 30.9% [95% Confidence Interval: 28.01–33.94%] in May 2019 to 62.5% [95% Confidence Interval: 59.38–65.6%] in September 2019 (See Fig. 5). The baseline VL testing coverage was 85% of eligible clients and the coverage following the intervention ranged between 94.8 and 98.8% in the 5 months report period. Of the VL test results received, the VL suppression rate ranged between 92.3 and 94.8% in the intervention period (baseline of 90%).

## Discussion

A twofold improvement in HIV testing, HIV case finding, and treatment initiation was demonstrated after 5 months of intervention. A similar improvement was seen in the 10 sub-cities for the three indicators except Akaki Kaliti sub-city where the improvement was relatively less in terms of HIV testing. The baseline performance in Akaki Kaliti sub-city for HIV testing was the lowest and the relatively poor achievement after the intervention is likely related with the lower baseline performance. The accelerated approach capitalized on Ethiopia’s recent strategic focus on revising methods and practice of health data management including advancing data analysis practice, promoting the culture of information use at the place of generation, and strengthening feedback systems [10]. As compared with the trend in the past, there was a relatively better engagement of the leadership and technical team at the regional, sub-city, and health facility level which largely contributed to the success of HIV program monitoring, data management, information use, and feedback systems at all levels. AACAHB leadership were leading the regular review meetings and their engagement in using HIV data for monitoring performance and strategic decision making influenced the regional technical teams and sub-city and health facility counterparts to adopt a culture of evidence-based decision making. Our approach follows recommendations to empower leadership to promote implementing and sustaining interventions to improve demand for and use of data [11].

The M&E framework was based on the existing HMIS platform, which simplified the approach and avoided duplication of efforts. The approach used the existing data management system and engaged health workers who were already leading the HMIS effort, which helped in strengthening data quality, information use, and the feedback mechanism at all levels. Similar health quality improvement initiatives in diverse settings worked within existing health information systems, structures, and processes and improved data generation, information use, and HIV care [12–14]. Sub-city and region level reviews enabled the team to identify issues related with coordination, availability of supplies, skill gaps, referral linkage, and administrative matters; these issues were directed to the responsible body for action.

Our approach emphasized using HIV data at health facility, sub-city, and regional levels to improve HIV services and quality of clinical care. The existing performance monitoring team at health facilities were instrumental in using the health facility data for weekly review and performance improvement. A similar intervention in Zambia helped staff identify weaknesses, develop interventions for improvement, mobilize resources, and strengthen support systems to improve ART and prevention of mother-to-child HIV transmission services [13]. In our intervention, major issues identified at the health facility and sub-city level were escalated to AACAHB for further attention. Establishing the web-based system and availability of weekly data helped in creating demand for HIV data, improving the frequency of reviews, and identifying gaps, and complementing the routine monthly HMIS data review. The progress report was presented via tables, graphs, and scoring that facilitated interpretation by the leadership and technical team. Our intervention supports findings of other studies in Ethiopia that suggested the importance of presenting data in a format understandable by health workers that facilitates data analytics and health information utilization [15–20]. Building the data analysis capacity of health workers and making data available in a usable format could also be helpful for future health data use interventions.

Standardizing the review mechanism at all levels was helpful in identifying gaps, addressing gaps, and reviewing project progress regularly. The health facility, sub-city, and city teams engaged for the HIV program are also responsible for other health programs, and their experience gained in the HIV program also could improve other public health initiatives. In Haiti, an HIV-related quality improvement intervention was later expanded to non-HIV services [21]. Previous studies showed low to medium use of health data in Ethiopia: one-fourth to two-thirds of health workers in studied health facilities were using HMIS data for health program planning and improvement [15–20]. With leadership support, capacity building, and a standardized approach, our intervention empowered health workers to use existing HIV data for decision making and program monitoring regularly. This approach will help the Ethiopian Ministry of Health to improve information use among leaders, health program managers, and health workers in alignment with the information revolution agenda in HSTP [10]. Health program and data management team worked closely together to develop the M&E activities to enhance data ownership and use. The HIV program team was actively engaged in the M&E activities that helped increase the demand for HIV data and enabled the use of data for clinical decision making. Collecting information directly linked to program data requirements and the ultimate evidence-based decision making could improve client outcomes [11].

Our accelerated approach improved data use at the health facility, sub-city, and regional levels. The successful implementation of the monitoring mechanism and the web-based system is due to the high level of commitment and engagement of relevant stakeholders, starting from the design phase. No matter how well-designed a monitoring system is, it is simply a tool, and its optimal utility is achieved only when its design is appropriately matched to both task and user, and its function is clear [22]. The efforts at the city, sub-city, and health facility levels were synchronized in terms of M&E and technical support. Implementing data-driven decision making has helped health offices and facility-based HIV teams to identify neglected areas of care that are key drivers of health outcomes, which enabled the HIV program to align with national guidelines, promote evidence-based decision making, engage stakeholders, share experiences, and effectively coordinate HIV/AIDS interventions. AACAHB has maintained the accelerated approach and the Ministry of Health also has expanded the approach to additional regions in Ethiopia in 2021 which is at its early stage of implementation.

HIV program performance improved because of our accelerated approach, strong M&E system, and additional program interventions. The limitation of our study is that we only presented the overall accomplishment in establishing standardized monitoring mechanism and data driven decision making but did not describe the details of HIV program interventions in the accelerated approach which could be presented in a dedicated report. The engagement of leaders and their level of support is described in a qualitative manner however the study did not use objective metric to measure the level of leadership engagement. Although the health facilities included in this report are relatively uniform in terms of patient load, human resources and logistics, the improvement might not solely be ascribed to the intervention alone as we have not controlled for other factors.

## Conclusions

Regular data driven HIV program review was institutionalized at city, sub-city and health facility levels which further improved HIV program performance. Adopting the national agenda of information revolution and using the existing HMIS resources, including data systems, human resources, and communication channels, facilitated the M&E efforts in our accelerated campaign and enabled efficient use of resources without effort duplication. Building the capacity of health workers on health data analysis, creating demand for data, and summarizing the data in a usable format are paramount to enhancing health data utilization. The review of HIV program performance via score-based approach helped in identifying health facilities with poor performance for timely intervention and enabled replication of the experiences of best-performing sites. Our accelerated approach and data use for HIV program improvement can be replicated in similar settings and diverse health programs.

## Data Availability

The dataset analyzed in the current study are available from the corresponding author on reasonable request.
